# Modification effect of fenofibrate therapy, a longitudinal epigenomic-wide methylation study of triglycerides levels in the GOLDN study

**DOI:** 10.1186/s12863-018-0643-6

**Published:** 2018-09-17

**Authors:** Runmin Wei, Yanyan Wu

**Affiliations:** 1 Molecular Biosciences and Bioengineering, University of Hawai’i at Mānoa, 1955 East-West Rd, Honolulu, HI 96822 USA; 2 Office of Public Health Studies, University of Hawai’i at Mānoa, 1960 East-West Rd, Biomed D-104F, Honolulu, HI 96822 USA

**Keywords:** Methylation, TG, Repeated measures, Mixed models, Interaction, GSEA

## Abstract

**Background:**

Identification of interactions between epigenetic factors and treatments might lead to personalized intervention of diseases. This paper aims to examine the modification effect of fenofibrate therapy on the association of methylation levels and fasting blood triglycerides (TG), and the related biological pathways among methylation sites.

**Results:**

Mixed-effects models were employed to assess pre- and posttreatment associations and drug modification effects simultaneously. Five cytosine-phosphate-guanine (CpG) sites were found to be associated with TG levels before and after the fenofibrate therapy: cg00574958, cg17058475, and cg01082498 on *CPT1A* gene, chromosome 11; cg03725309 on *SARS*, chromosome 1; and cg06500161 on *ABCG1*, chromosome 21. In addition, fenofibrate therapy modified the methylation levels on the following 4 CpG sites: cg20015535 (gene *EGLN1*, chromosome 1); cg24870738 (gene *RNF220*, chromosome 1); cg06891775 (gene *LOC283050*, chromosome 10); and cg00607630 (gene *USP7*, chromosome 16). Further, gene set enrichment analysis (GSEA) identified cancer- and metabolism-related pathways that were associated with TG-related CpG sites.

**Conclusions:**

We identified modification effects of fenofibrate on the associations between blood TG levels and several CpG sites. Pathway enrichment analysis indicated the alternations in some metabolism and cancer-related pathways. Our findings have important implications for future research in pharmacoepigenetics and personalized medicine.

## Background

Large-scale genome-wide association studies (GWAS) have identified numerous loci associated with fasting blood lipids and other cardiovascular diseases (CVDs) [1]. Epigenetic analysis has gained attention in the past few years as an alternative perspective on the etiology of complex diseases. Epigenetic adaptations alter gene expressions and are heritable through many cell divisions, even across generations, while they do not alter the primary DNA sequence. To advance blood lipids and CVD research, it is important to apply epigenome-wide association study (EWAS) to detect the epigenetic risk factors. The study of molecular mechanisms underlying epigenetic inheritance, such as DNA methylation, will provide insights in advancing and shaping ideas of the role that epigenetic phenomena play in high blood lipids and CVD. In this paper, we used data from the Genetics of Lipid-lowering Drugs and Diet Network (GOLDN) study provided by the GAW20 to examine the methylation levels of lipid-lowering treatment on the fasting blood triglycerides [2].

## Methods

### Data

The GAW20 data sets are drawn from the GOLDN study with a total number of 1105 participants [2]. The data sets include GWAS and EWAS data before and after the fenofibrate (blood lipid-lowering drug) intervention. The EWAS data set contains 2 triglyceride (TG) measurements and methylation levels of 463,995 cytosine-phosphate-guanine (CpG) sites for 995 pretreatment individuals and 530 posttreatment individuals, respectively. The log-transformed mean pre- and posttreatment TG levels were used as the outcome variable in our model. Control variables include age, gender, study center, and family pedigree.

### EWAS model

We applied mixed-effects models for two repeated measures of log TG levels with fixed effects of time (0 = pre, 1 = post), methylation level, and their interactions, adjusting for age (18 years of age to approximately 87 years of age), sex, study site, and top 4 methylation principal components. Pedigree and subject IDs are controlled as nested random effects. These fixed effects of time, methylation levels, and the interaction term, measure the associations for both pre- and posttreatment periods, and the treatment modification effects, respectively.

Let *Y*_*ijk*_ denote the log TG measurements at *k*th time (0 = pre, 1 = post) for the *i*^th^ individual in the *j*^th^ pedigree; *X*_*ijk*_ denote the methylation level; and *t*_*k*_ denote treatment while *t*_0_= 0 and *t*_1_=1. The model equation can be written as:

$$ {\displaystyle \begin{array}{l}{Y}_{ij k}={\beta}_0+{\beta}_1{X}_{ij k}+\gamma {t}_k+\delta \left({X}_{ij k}{t}_k\right)+{\beta}_2{Age}_i+{\beta}_3{Site}_i\\ {}+{\beta}_4 PC{1}_{ik}+{\beta}_5 PC{2}_{ik}+{\beta}_6 PC{3}_{ik}+{\beta}_7 PC{4}_{ik}\\ {}+{S}_{ij}+{\varepsilon}_{ij k}\end{array}} $$

where the main effect *β*_1_is the pretreatment methylation effect on log TG; *γ* is the main treatment effect; δ is the interaction effect between methylation and treatment (i.e., the treatment modification effect); and *S*_*ij*_ is the random effect of the individual nested within the pedigree. The general linear hypothesis tests were applied to calculate postmethylation effect (*β*_1_ + *δ*), the standard errors, and the *p* values. We examined each CpG site on the whole genome (463,995 sites). Mixed-effects models for repeated measures enable us to examine the individual patterns of change by excluding between-individual variability and provide more efficient estimators of treatment effects. The main effects and interactions work together to identify the epigenetic risk factors of TG levels for pretreatment, posttreatment, and potential gene–drug interactions simultaneously [3, 4]. Compared to cross-sectional study, the repeated measure analysis has the advantage of making reliable inferences by capturing the systemic changes within individuals, thereby achieving more sensitive tests and higher statistical power for a fixed number of individuals [5, 6]. Statistical software R (version 3.2.3) was used for the entire analyses, with R package *nlme* for mixed-effects modeling [7], *car* for linear hypothesis tests [8]*,* and *qqman* for Manhattan plots [9].We applied a relatively loose significance threshold (*p* value <1E-5) for modification effects and posttreatment associations because of the exploratory nature of proposed method and the moderate sample size (*N* = 536 posttreatment measures). A less-stringent threshold might imply potential drug modification effects, as empirical evaluation suggests a possible relaxation in the current GWAS threshold for replication studies [10].

### Pathway-enrichment analysis

After EWAS analyses of CpG sites for pretreatment, posttreatment, and interaction effects, we mapped them to corresponding genes. To provide a functional insight of the results, we applied a gene set enrichment analysis (GSEA) [11] preranked test to each of 3 gene lists with log-transformed *p* values. To compute the empirical *p* values and false discovery rates (FDRs) for pathways, we performed 1000 permutations. Pathways from the Kyoto Encyclopedia of Genes and Genomes (KEGG) database [12] were used in our analysis.

GSEA is a robust technique that searches for pathways (gene sets) that contain abundant highly significant genes (CpG sites) based on a Kolmogorov-Smirnov test [11] to reveal biological insights of genome/epigenome data.

## Results

**Fig. 1:**
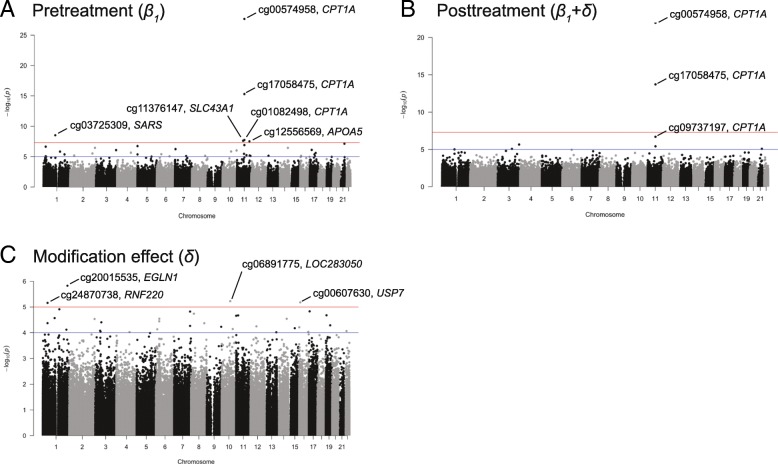
Manhattan plots of EWAS analyses for (**a**) pretreatment, (**b**) posttreatment, (**c**) and drug–gene interactions (i.e., drug modification effect)

Table 1 lists selected CpG sites that are associated with pre- and post-log TGs, and modified by treatment, and Fig. 1 shows the corresponding Manhattan plots. The methylation level of 3 CpG sites (cg00574958, cg17058475, and cg01082498) on *CPT1A* gene, chromosome 11, and 2 other CpG sites (cg03725309 on gene *SARS*, chromosome 1, and cg06500161 on *ABCG1*, chromosome 21) were found to be associated with both pre- and posttreatment TG levels (*p* values <1E-5). Moreover, the methylation levels of 2 sites on chromosome 11 are associated with pretreatment log TG but not with posttreatment log TG levels (cg12556569 on gene *APOA5* and cg11376147 on gene *SLC43A1*).

**Table 1:** Selected EWAS CpG sites for pretreatment, posttreatment, and gene–drug interactions (*p* value <1E-5 is considered statistical signifiance and italicized)

CpG site	Chr	Location	Genes	Pretreatment *β*_*1*_ (95% CI)	Posttreatment *β*_*1*_ + *δ* (95% CI)	Modification effect (95% CI)	*p* value (Pre-)	*p* value (Post-)	*p* value (Modif.)
*Significant effect modification sites*									
cg20015535	1	231,556,123	*EGLN1*	1.30 (0.11, 2.49)	−3.1 (−4.77, −1.43)	− 4.4 (−6.17, − 2.63)	3.31E-02	2.80E-04	*1.49E-06*
cg24870738	1	45,097,499	*RNF220*	−3.8 (−5.9, −1.70)	2.23 (0.58, 3.88)	6.03 (3.44, 8.62)	4.40E-04	7.81E-03	*6.90E-06*
cg06891775	10	80,704,982	*LOC283050*	2.13 (1.28, 2.98)	0.52 (−0.29, 1.33)	−1.61 (−2.3, − 0.92)	*1.39E-06*	2.10E-01	*5.91E-06*
cg00607630	16	9,030,093	*USP7*	2.82 (1.61, 4.03)	0.04 (−1.15, 1.23)	−2.78 (−3.97, −1.59)	*7.18E-06*	9.53E-01	*6.60E-06*
*Significant pre or post methylation sites*									
cg03725309	1	109,757,585	*SARS*	−1.65 (−2.18, − 1.12)	− 1.28 (− 1.85, −0.71)	0.37 (0.04, 0.7)	*3.00E-09*	*9.82E-06*	2.50E-02
cg12556569	11	116,664,039	*APOA5*	0.35 (0.23, 0.47)	0.23 (0.08, 0.38)	−0.12 (−0.24, 0)	*3.11E-08*	2.34E-03	4.56E-02
cg11376147	11	57,261,198	*SLC43A1*	−2.24 (−3.01, −1.47)	−1.46 (− 2.37, −0.55)	0.78 (0, 1.56)	*2.46E-08*	1.60E-03	5.20E-02
cg00574958	11	68,607,622	*CPT1A*	−3.81 (−4.44, − 3.18)	− 3.55 (−4.26, −2.84)	0.26 (−0.31, 0.83)	*2.12E-28*	*1.07E-22*	3.65E-01
cg01082498	11	68,608,225	*CPT1A*	−2.59 (−3.48, −1.7)	−2.38 (−3.39, − 1.37)	0.21 (−0.66, 1.08)	*1.86E-08*	*3.83E-06*	6.30E-01
cg17058475	11	68,607,737	*CPT1A*	−2.31 (−2.84, −1.78)	−2.34 (− 2.94, − 1.74)	−0.04 (−0.45, 0.37)	*5.00E-16*	*1.91E-14*	8.63E-01
cg06500161	21	43,656,587	*ABCG1*	2.26 (1.45, 3.07)	2.16 (1.21, 3.11)	−0.09 (−0.86, 0.68)	*7.55E-08*	*8.18E-06*	8.16E-01

In addition, the associations between methylation level and log TG levels were modified by the fenofibrate therapy on the following four CpG sites with *p value* < 1E-5:cg20015535 (gene *EGLN1*, chromosome 1), cg24870738 (gene *RNF220*, chromosome 1), cg06891775 (*gene LOC283050*, chromosome 10), and cg00607630 (gene *USP7*, chromosome 16).

The GSEA results are recorded in Table 2, which shows FDR *q-*values for the top 15 pathways across pre- and posttreatment associations, and the treatment-modifying effects. Several cancer-related pathways (KEGG_ENDOMETRIAL_CANCER; KEGG_PATHWAYS_IN_CANCER; KEGG_CHRONIC_MYELOID_LEUKEMIA; KEGG_BASAL_CELL_CARCINOMA; and KEGG_NON_SMALL_CELL_LUNG_CANCER) show consistent significant results. In addition, metabolism related pathways were also observed (KEGG_TYPE_II_DIABETES_MELLITUS and KEGG_ADIPOCYTOKINE_SIGNALING_PATHWAY).

**Table 2:** FDR *q*-values for the top 15 pathways across different results from GSEA

Pathway name	Pretreatment FDR *q* value	Posttreatment FDR *q* value	Modification FDR *q* value
KEGG_ENDOMETRIAL_CANCER	0.146	0.023	0.040
KEGG_PATHWAYS_IN_CANCER	0.166	0.059	0.137
KEGG_DORSO_VENTRAL_AXIS_FORMATION	0.182	0.067	0.122
KEGG_ADHERENS_JUNCTION	0.141	0.079	0.139
KEGG_TYPE_II_DIABETES_MELLITUS	0.198	0.079	0.092
KEGG_COLORECTAL_CANCER	0.205	0.078	0.107
KEGG_ADIPOCYTOKINE_SIGNALING_PATHWAY	0.160	0.081	0.138
KEGG_FOCAL_ADHESION	0.165	0.061	0.153
KEGG_MELANOGENESIS	0.178	0.077	0.139
KEGG_ALDOSTERONE_REGULATED_SODIUM_REABSORPTION	0.193	0.017	0.156
KEGG_CHRONIC_MYELOID_LEUKEMIA	0.160	0.134	0.133
KEGG_BASAL_CELL_CARCINOMA	0.153	0.055	0.199
KEGG_CALCIUM_SIGNALING_PATHWAY	0.236	0.067	0.133
KEGG_WNT_SIGNALING_PATHWAY	0.230	0.075	0.133
KEGG_NON_SMALL_CELL_LUNG_CANCER	0.289	0.070	0.109

## Discussion

The proposed mixed-effects model examines the methylation sites that are associated with blood TG levels before and after the fenofibrate therapy, and the potential gene–drug interactions. Using the linear hypothesis test, we identified 7 CpG sites that are associated with pretreatment TG levels (*p* value <1E-7) and 5 sites for posttreatment (*p* value <1E-5). All 5 posttreatment CpG sites are overlapped with pretreatment CpG sites. Among these CpG sites, 3 are located in gene *CPT1A*, which encodes a key enzyme in the carnitine-dependent transport of long-chain fatty acids across the mitochondria membrane whose deficiency will result in downregulation of fatty acid *β*-oxidation [13].The consistent findings suggest a strong association between blood TGs and DNA methylation of *CPT1A* regardless of the interference of lipid-lowering drug. In addition, we also observed 4 potential drug-interacted CpG sites from our results that belong to genes *EGLN1*, *LOC283050*, *USP7*, and *RNF220*. Previous study shows that the inhibition of *EGLN1* improves the glucose and lipid metabolism, and protects against obesity and metabolic dysfunction [14]. Less-significant results were observed for drug modification effects, which were in part a result of the moderate sample size (536 posttreatment measures). But our results provide initial evidence of gene–drug interaction and warrant replication studies [10].

To provide further biological insight to 4 EWAS results, GSEA was applied to examine the associated biological pathways using KEGG database [12]. It is worth noting that 5 cancer-related pathways were enriched by TG-associated CpG sites. We observed potential associations between blood TG levels and cancer risk from an epigenetic point of view. Although obesity was recognized as a risk factor for several different cancers, for example, endometrial cancer [15], further mechanism research is necessary to determine whether there is any association between methylation level and cancer risk. In addition to these cancer-related pathways, 2 metabolism-related pathways were also observed. For Type II diabetes, elevated TG levels are common dyslipidemic features [16] and could be identified as an independent risk factor [17]. Another significant pathway is the KEGG_ADIPOCYTOKINE_SIGNALING_PATHWAY. In addition to the fatty acid metabolism and *β*-oxidation, this pathway is also associated with glucose uptake and insulin resistance.

## Conclusions

In summary, we used linear mixed models with interaction terms to study pre- and posttreatment associations between blood TGs and CpG methylation levels and drug–gene interactions simultaneously across the whole-genome. We found several CpG sites that were consistently associated with blood TG levels in both pre- and posttreatment. In addition, by testing on the interaction term, we found potential treatment modification effects on certain CpG sites. Our pathway-enrichment analysis revealed a number of cancer-related biological pathways that were significantly enriched by TG-associated CpG sties. The results suggest connections between TG levels and cancer risk through an epigenetic point of view. However, because only 1 cohort with a limited sample size was studied in our analyses, further research on independent cohorts and experimental biology validations are needed for convincing conclusions.

## Abbreviations

CpG: Cytosine-phosphate-guanine
CVDs: Cardiovascular diseases
EWAS: Epigenome-wide association study
FDRs: False discovery rates
GAW: Genetic Analysis Workshop
GOLDN: Genetics of Lipid-lowering Drugs and Diet Network
GSEA: Gene set enrichment analysis
GWAS: Genome-wide association studies
KEGG: Kyoto Encyclopedia of Genes and Genomes
TG: Triglycerides
